# Mapping human disease-associated enzymes into Reactome allows characterization of disease groups and their interactions

**DOI:** 10.1038/s41598-022-22818-5

**Published:** 2022-10-26

**Authors:** Castrense Savojardo, Davide Baldazzi, Giulia Babbi, Pier Luigi Martelli, Rita Casadio

**Affiliations:** 1 Biocomputing Group, Department of Pharmacy and Biotechnology, University of Bologna, Bologna, Italy; 2 CRO, Centro di Riferimento Oncologico, Aviano, Italy; 3 Institute of Biomembranes, Bioenergetics and Molecular Biotechnologies (IBIOM), Italian National Research Council (CNR), Bari, Italy

**Keywords:** Computational biology and bioinformatics, Data integration, Databases

## Abstract

According to databases such as OMIM, Humsavar, Clinvar and Monarch, 1494 human enzymes are presently associated to 2539 genetic diseases, 75% of which are rare (with an Orphanet code). The Mondo ontology initiative allows a standardization of the disease name into specific codes, making it possible a computational association between genes, variants, diseases, and their effects on biological processes. Here, we tackle the problem of which biological processes enzymes can affect when the protein variant is disease-associated. We adopt Reactome to describe human biological processes, and by mapping disease-associated enzymes in the Reactome pathways, we establish a Reactome-disease association. This allows a novel categorization of human monogenic and polygenic diseases based on Reactome pathways and reactions. Our analysis aims at dissecting the complexity of the human genetic disease universe, highlighting all the possible links within diseases and Reactome pathways. The novel mapping helps understanding the biochemical/molecular biology of the disease and allows a direct glimpse on the present knowledge of other molecules involved. This is useful for a complete overview of the disease molecular mechanism/s and for planning future investigations. Data are collected in DAR, a database that is free for search and available at https://dar.biocomp.unibo.it.

## Introduction

Increasing evidence supports the notion that mutations in human genes might lead to loss/gain of functions of proteins. Genetic diseases and associated genes are traditionally listed in OMIM (https://www.omim.org/). Nowadays, it is known that a large fraction of the OMIM genetic disease collection is associated with enzyme deficiencies^1–4^. In the presence of enzyme deficiencies, both substrate concentration and/or loss in metabolites due to enzyme-catalyzed reactions may alter the overall cell steady state, causing pathologies ranging from mild to severe ones^5^. When interpreting the effect of a given pathology, elicited by the presence of enzyme variants in the human cell, it is necessary to know which biochemical pathways and reactions are involved.

It is common knowledge that each single protein/enzyme is well annotated in its UniProt/SwissProt file (https://www.uniprot.org/), which links different sources of information, ranging from structural ones, when present, to functional ones. For disease-related proteins, relevant diseases, including the genetic ones, and the associated germline variations are listed, when reported in databases and/or literature.

For a better understanding of the variant effect, it is important to focus which biological process/es is/are involved and which other molecules are in the neighborhood of the targeted one. For enzymes, this is particularly important if we think that these molecules can also modulate the concentration of all the necessary metabolites in the biological processes they co-participate.

The inherent structural and functional complexity of the cell interior can be described in terms of biological pathways, which enclose all the concomitant reactions. Major descriptors of this complexity are biological processes with focus on biochemical reactions (with substrate and product molecules), such as in KEGG (The Kyoto Encyclopedia of Genes and Genomes, https://www.genome.jp/kegg/) and in Reactome (https://reactome.org/). Here we adopt Reactome, given its inherent hierarchical architecture that allows clustering of different biochemical reactions into subgroups and groups of pathways, sampling most of the different aspects of the cell metabolism.

In this study, we are interested in exploiting to what extent enzymes variants related to genetic diseases affect cell biological processes and particularly metabolism. To this aim, we first map enzymes with known disease-associations on the Reactome universe in relation to their catalytic activity and highlight enzyme-Reactome links. Then by grouping diseases according to the corresponding Reactome tree, we derive a major categorization of human genetic diseases elicited by enzyme variants. By this, we can distinguish four major types of categories (with few subtypes) of monogenic and polygenic diseases. All our data are available in the accompanying DAR (Disease And Reactome) database, which is freely browsable, providing an exhaustive knowledge of the disease-associated enzyme at hand and its molecular and functional environment.

## Materials and methods

### Disease data collection

Human genes were retrieved from the UniProt Reference proteome (https://www.uniprot.org/proteomes/UP000005640, release 2022_01), listing 20,598 genes mapped on the corresponding primary isoforms. For these genes, we extracted disease associations from three resources: the UniProt database (using the Pathology & Biotech field of each entry), the Monarch initiative database (https://monarchinitiative.org, as in March 2022) and ClinVar (https://www.ncbi.nlm.nih.gov/clinvar/, as in March 2022) We excluded all associations marked as somatic. From UniProtKB we obtained 5656 diseases associated with 4276 genes in the reference proteome. From Monarch, we retrieved 6213 diseases mapped on 4137 genes in our set. ClinVar contributed with 3197 genes with 4629 annotated diseases. After performing a manual and rational merging of these associations from the three databases (taking care of resolving all the inconsistencies related to gene/protein nomenclature) we obtained 4650 genes associated with 7023 diseases. We refer to this dataset as the Union set.

Enzymes in the Union set were identified by selecting proteins having an associated Enzyme Commission (EC) number and/or a Gene Ontology (GO) Molecular Function term which is a descendant of “GO:0,003,824 – catalytic activity”. To compute GO descendancy we used an in-house script processing the GO OBO file (release 2022–05-16). After this procedure, we restricted our Union set to 1494 human enzymes associated with 2539 genetic diseases.

All diseases were mapped on the corresponding MONDO disease ontology identifier (OBO release 2022–03-01).

### Reactome

Reactome lists, for *Homo sapiens*, a total of 2580 pathways hierarchically organized into 29 main roots, 737 internal nodes and 1814 leaf pathways (including the catalyzed reaction/s) (Version 80, released 05 April 2022, https://reactome.org/about/news/175-version-80-released). Out of these, 1528 pathways (including 960 leaf-pathways, 541 internal nodes, 27 roots) are associated in the UniProt protein files with the 1494 disease-related enzymes, functionally annotated with major variations, when present, and associated to specific genetic diseases, included in the Mondo disease ontology (https://www.ebi.ac.uk/ols/ontologies/mondo).

The 27 Reactome hierarchical roots exclude Disease, which includes only a small fraction (324 enzymes) of our set of disease-related enzymes, and Drug ADME, which includes disposition of pharmaceutical compounds within an organism (described by their four main stages: Absorption, Distribution, Metabolism, and Excretion).

### The DAR database

The Disease And Reactome (DAR) database is available at https://dar.biocomp.unibo.it. The database is stored on a backend PostgreSQL DBMS (v.11.14; https://www.postgresql.org). The server-side web application is implemented using the Django Python application server (v.4.0.4, https://www.djangoproject.com), while the user interface adopts the Bootstrap framework (v5, https://getbootstrap.com), JQuery (v3.6, https://jquery.com) and DataTables (v1.12, https://datatables.net).

From the web interface, the user can interrogate the database directly by selecting the type of query to perform and filling the search box. The database can be searched for: (1) UniProt Accession, (2) Gene Name, (3) Disease ID (either MONDO, OMIM or Orphanet), (4) Disease Name, (5) Reactome pathway (either ID or name), (6) Reactome root pathway and 9) EC number (either 4- or 1-digit EC). All the queries support autocompletion, to help the user properly selecting the input values.

The user can choose to explore the database content by browsing DAR by different entities, including diseases, Reactome pathways, enzymes and their expression in the different tissues, and EC numbers.

Upon query submission, DAR returns to the user a page containing a summary table and the data organized in a tabular format. Each row reports a disease-enzyme association alongside several additional information, including EC numbers, Reactome pathways (both those directly annotated on the gene/protein and those derived from the expansion on the complete Reactome tree), Reactome Roots where the annotated pathways map to, and all Reactions (from Reactome) associated to the enzymes. From the table, the user can retrieve details about these fields and the links to reference resources: Monarch/OMIM/Orphanet for diseases, UniProtKB for enzyme genes/proteins and Reactome web site for pathways and reactions.

We plan to update DAR every year, including the latest releases of all the databases included.

## Results

A major issue in gene variant functional annotation is the knowledge of the available information, which is often sparse in different resources. More to this, it is difficult to retrieve reliable data with experimental documentation that describes a disease-gene variant association and variants effecting the biochemistry of the cell. We tackle this problem by considering as a reference description of the cell space the human Reactome pathways and their hierarchical organization.

In the following, we will comment on how we perform the mapping of human genetic diseases elicited by enzyme known germline variants on the different human Reactome pathways, and highlight the most interesting results in terms of the associations that we produce and collect in DAR, the database of Diseases and Reactome pathways associations. Enzymes associated to human genetic diseases have a central role in determining the association.

### Statistics of DAR

**Figure 1:**
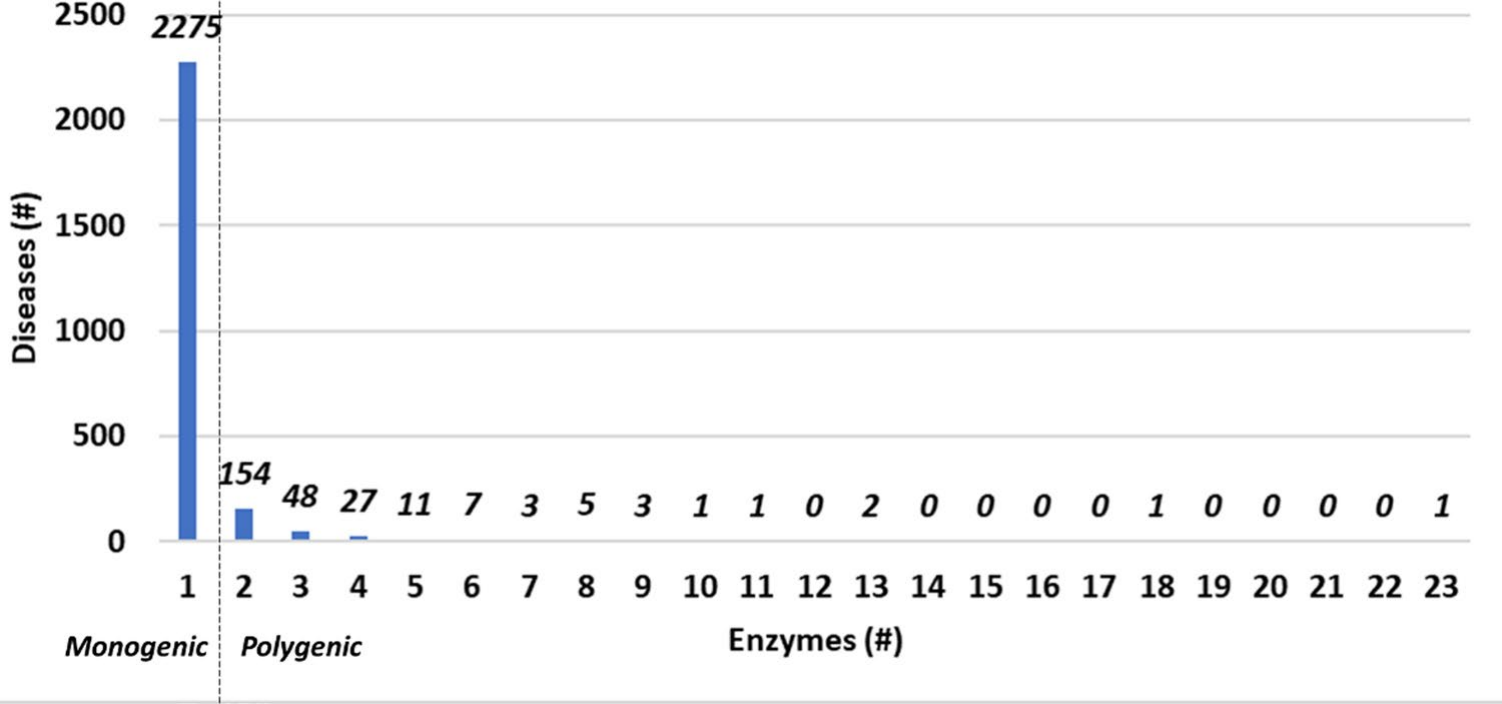
Distribution of diseases according to the number of enzymes they are associated to. #: number of. The number of diseases is reported at the top of each bar. 2275 diseases are associated to only one enzyme. Out of these, 300 are associated to other, non-enzyme, proteins (data not shown). The tail of the distribution comprises one disease (“intellectual disability”, MONDO:0,001,071) associated with 23 enzymes and one disease (“colorectal cancer”, MONDO: 0,005,575) associated with 18 enzymes.

The DAR database presently contains 1496 enzymes. They represent 9% of the total enzyme content of the human reference proteome (https://www.uniprot.org/proteomes/), and 27.6% of the human enzyme content in SwissProt, which includes protein records with curator-evaluated information and computational analysis (Fig. 1S). These enzymes are associated to 2539 human genetic diseases with 3108 associations (derived from Humsavar, https://www.uniprot.org/docs/humsavar.txt; OMIM, https://www.omim.org/; ClinVar, https://www.ncbi.nlm.nih.gov/clinvar/; and Monarch, https://monarchinitiative.org/).

Enzyme-disease associations are biunivocal (bijective, one enzyme associated to only one disease and the disease associated only to that enzyme), univocal (one enzyme associated to one disease and the disease associated also to other enzyme/s) and non-univocal ( one enzyme associated to more than one disease) (Table 1). The classification of the types of enzyme-disease associations provides a general description of the selected database.

**Table 1:** Dataset description.

Set	Enzymes	Diseases *	Associations	Reactome annotations ^	Reactome Roots °
Biunivocal enzyme to disease relations	744	744	744	805 (1167; *2798*)	27
Univocal enzyme to disease relations	87	61	87	212 (424; *513*)	20
Non univocal enzyme to disease relations	663	1793	2277	949 (1304; *3845*)	27
Total	1494	2539	3108	1165 (1528; *5570*)	27

* The same disease can appear in more than one list, ^ the same Reactome term can appear in more than one list; values between brackets indicate the expanded Reactome list from leaves to roots and the reaction numbers (in italic), respectively; ° the same Reactome root can appear in more than one list.

Reactome Roots collect all the specific Reactome pathways in a hierarchical organization (https://reactome.org)^6^. Disease-related enzymes map in 1528 of the Reactome pathways, included in 27 Reactome hierarchical roots.

Reactome leaves include for each enzyme the specific reaction/s which it contributes with reactants and products to the cell biological processes. When variations hamper the protein molecular function, the pathway is affected. In addition, all the related pathways can be perturbated. Enzymes map to Reactome pathway/s and the main information is the specific reaction/s that the enzyme catalyses. The Reactome hierarchical organization allows to group leaves and intermediate nodes into a root, for sake of generalization.

Enzymes are routinely described by considering the Enzyme Commission number which classifies them according to seven major classes (Table 2, Fig. 1S). Presently, enzyme classes contributing the most to disease-enzyme associations are Transferases (EC.2), Hydrolases (EC.3) and Oxidoreductases (EC.1) (Fig. 1S).

**Table 2:** Enzyme classification.

Set	Enzymes	Diseases *	Associations	Reactome annotations ^	Reactome Roots °
EC 1: Oxidoreductases	208	334	361	203	20
EC 2: Transferases	544	1023	1212	801	26
EC 3: Hydrolases	445	868	960	640	27
EC 4: Lyases	52	77	77	62	14
EC 5: Isomerases	34	68	72	65	16
EC 6: Ligases	63	99	102	32	7
EC 7: Transferases	56	110	142	34	11
Multiclass ^$^	55	120	124	118	15
Without EC ^§^	37	75	80	46	9
Total	1494	2539	3108	1165	27

* The same disease can appear in more than one list, ^ the same Reactome term can appear in more than one list; ° the same Reactome root can appear in more than one list. ^$^ Multiclass: enzymes endowed with two or more EC level 1 classifications. ^§^ Without EC: enzymes annotated with the GO term “Catalytic activity” and not endowed with an EC classification.

**Figure 2:**
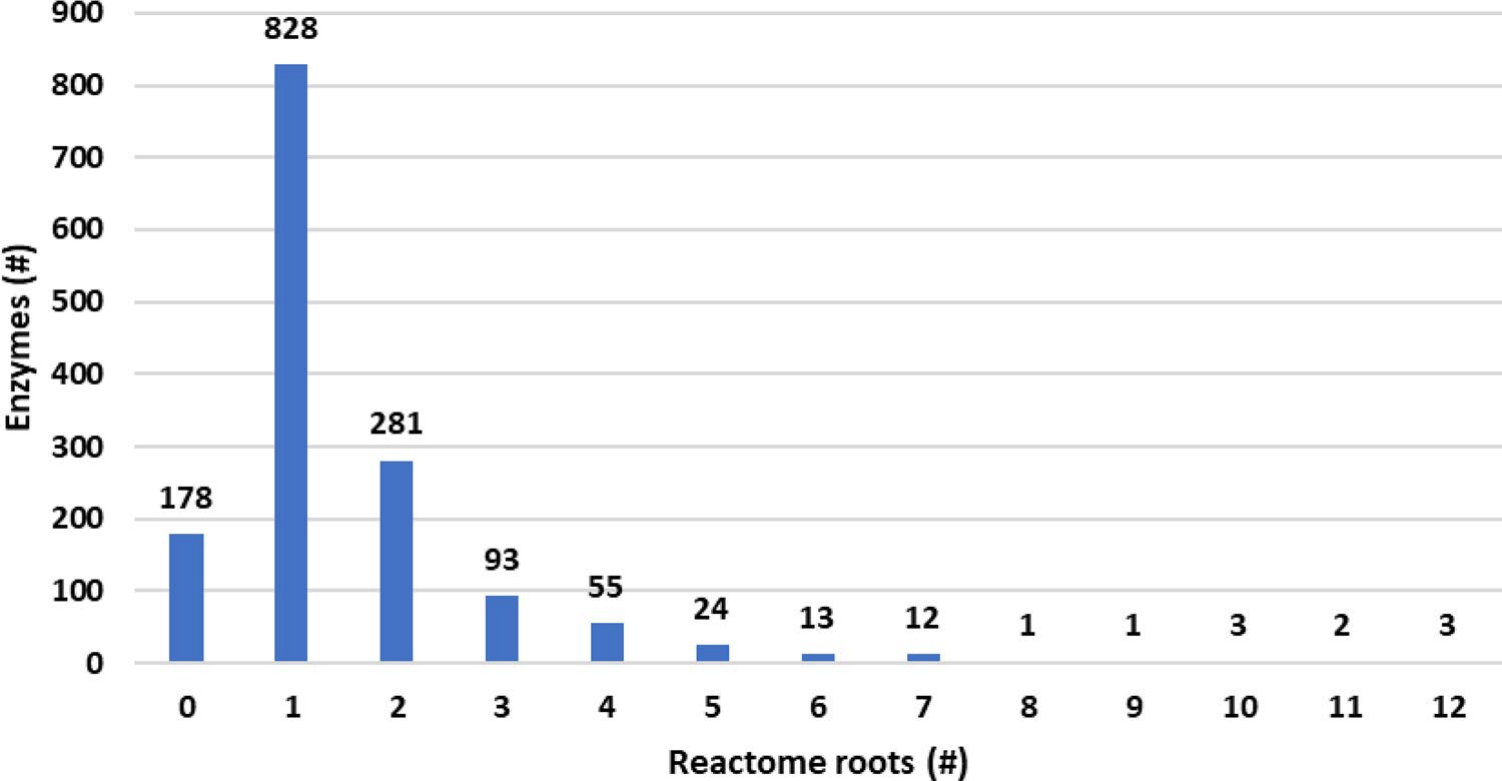
Distribution of enzymes according to the number of Reactome roots they belong to. #: number of. The number of enzymes in each group is reported at the top of each bar. Roots “Disease” (R-HSA-1643685) and "Drug ADME" (R-HSA-9748784) are not considered. Enzymes associated to 12 different Reactome roots are three subunits of the proteasome (Proteasome subunit beta type-10, P40306; Proteasome subunit beta type-9, P28065; Proteasome subunit beta type-8, P28062).

Figure 1 shows the disease distribution as a function of the number of associated enzymes. 2275 diseases are monogenic being associated to only one enzyme. The remaining ones are polygenic, with two diseases, intellectual disability and colorectal cancer, associated to 23 and 18 enzymes, respectively. Alternatively, Fig. 2 highlights the distribution of the disease-associated enzymes as a function of the Reactome main roots. 55% of the DAR enzyme content is associated to only one Reactome root, while the remaining part is differently distributed in more than one root. As an extreme case, three subunits of the proteasome are included in 12 of the 27 Reactome roots, here considered.

### DAR and the complexity of the enzyme-disease-Reactome mapping

Genetic diseases are classically categorized as monogenic (associated with only one gene), polygenic (associated with more than one gene) and chromosomal syndromes/abnormalities^7,8^. Considering genes and diseases, the association is rather complicated (see also Fig. 2S). When we focus on the disease-enzyme-Reactome association we can dissect this interaction-space and derive useful categorizations detailed in the following.

### Clustering of monogenic and polygenic diseases

**Figure 3:**
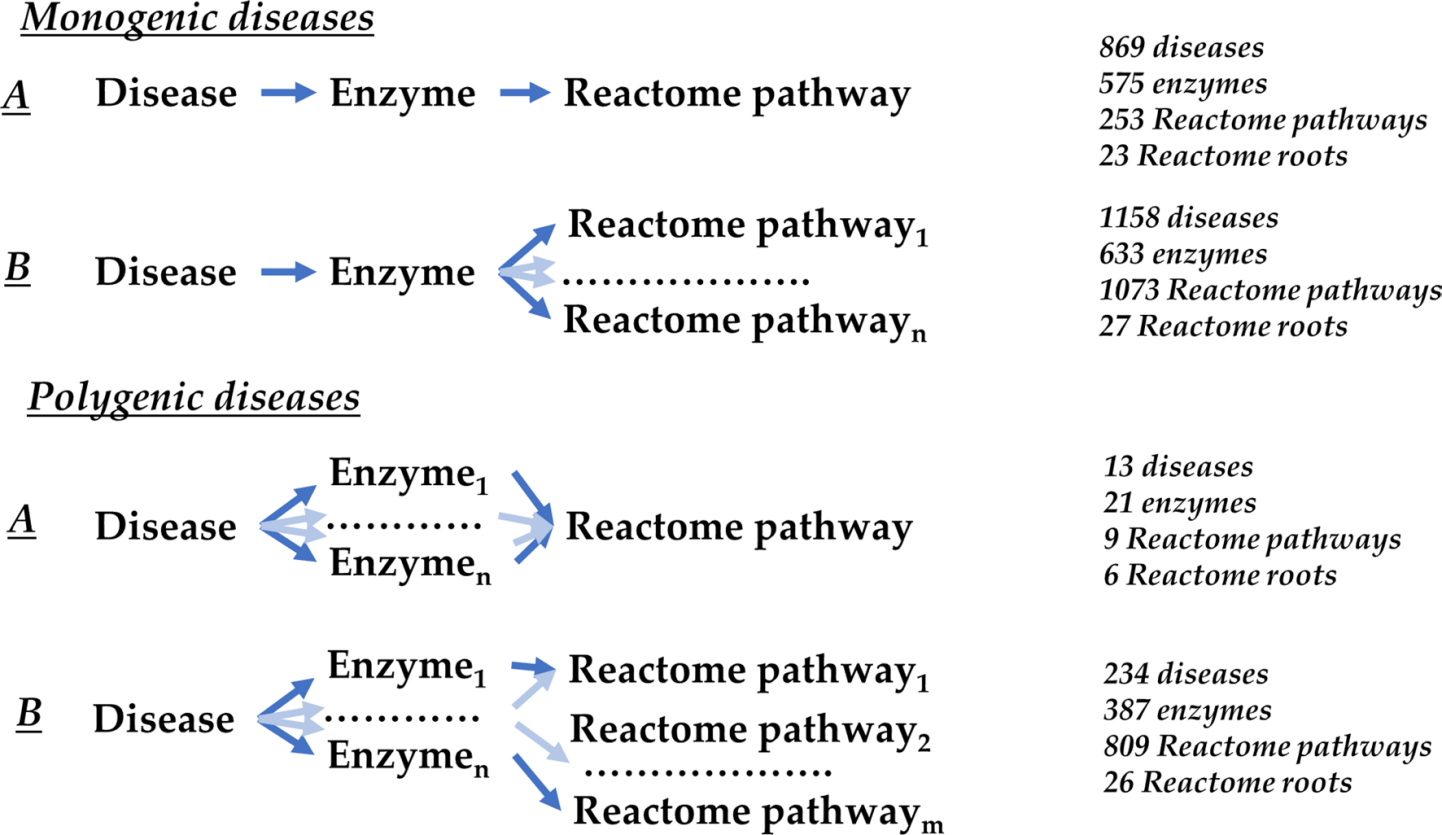
Relations among diseases and Reactome pathways, as mediated by the enzymes included in DAR. The total number of diseases is 2539. 178 Enzymes out of 1494 lack Reactome annotation. Therefore, 265 diseases (248 monogenic and 17 polygenic) are excluded from the figure.

The distribution of monogenic and polygenic diseases after disease-enzymes mapping into Reactome allows four main categories (Fig. 3).

Monogenic diseases are associated with one enzyme, however this enzyme can be involved into one Reactome pathway (type A) or into more than one Reactome pathways (type B); polygenic diseases can include enzymes with a unique Reactome mapping (type A) or with plurivocal Reactome mappings (type B).

### Monogenic diseases in DAR

The large majority of diseases (2275, see Fig. 1) collected in DAR is associated with one single enzyme (1683 of these are “rare”, with an Orphanet code, https://www.orpha.net/consor4.01). Only in 300 cases, associations between the same diseases and other non-enzyme proteins are reported in databases.

The 869 type A monogenic diseases (Fig. 3) are associated with a single Reactome pathway and this allow to further group diseases that share the same pathways. However, based on the associations among diseases, genes, and pathways, different monogenic patterns are possible (as described in Fig. 3S).

Even if the relation between disease and gene seems to be straightforward, a defect on an enzyme can end up in a complex effect at the physiological level. Indeed, 1158 monogenic diseases in DAR are linked to 633 enzymes that participate in more than one Reactome pathway (Fig. 3, Monogenic disease-B). The most extreme case is the Proto-oncogene tyrosine-protein kinase Src (SRC, UniProt: P12931, EC:2.7.10.2) that is involved in 55 different Reactome pathways, belonging to 6 different roots (primarily Signal transduction, then Autophagy, Cell–cell communication, Developmental biology, Hemostasis, Immune system). Such a polymorphic role in the cellular pathways can explain the diversity of the two monogenic diseases associated with SRC, namely osteoporosis (Mondo:0005298) and thrombocytopenia 6 (Mondo:0014837).

Osteoporosis is a disorder characterized by the deterioration of the bone tissue, and mainly due to the impairment of bone homeostasis, where SRC plays a central role (it promotes bone resorption by osteoclasts and suppresses bone formation by osteoblasts^9^), as it is also described in the Reactome pathway R-HAS-8940973 (RUNX2 regulates osteoblast differentiation).

Thrombocytopenia-6 is a hematologic disorder characterized by a reduced platelet count and abnormal platelet morphology resulting from defective megakaryopoiesis. It is caused by a gain-of-function missense variation of SRC (E527K) that lead to the constitutive activation of the enzyme^10^, perturbating two Reactome pathways in the Hemostasis root (R-HAS-210291: Phosphorylation of PECAM-1 by Fyn or Lyn or c-Src; R-HAS-443418: GP1b signaling involves c-Src). Interestingly, patients carrying the variation exhibit comorbidities, in particular (polygenic) diseases, such as myelofibrosis and bone pathologies, which are reported in DAR.

The participation of a single enzyme in many different pathways can help in understanding the multiplicity of symptoms or phenotypes associated with a specific disease^11^. This is the case of three Proteasome-Associated Autoinflammatory Syndromes (PRAAS1 (Mondo:0054698), PRAAS3 (Mondo:0054699), and PRAAS5 (Mondo:0030924)) caused by variations on three proteasome subunits (subunit beta type-8 (P28062), subunit beta type-9 (P28065), and subunit beta type-10 (P40306), respectively). The three proteins have EC:3.4.25.1 and participate in 53 Reactome leaf-pathways each. With few exceptions, pathways are common to all the three enzymes and encompass 12 different Reactome roots (Cell-cycle, Cellular responses to stimuli, DNA replication, Developmental biology, Gene expression, Immune system, Metabolism, Metabolism of DNA, Metabolism of proteins, Programmed cell death, Signal transduction, Transport of small molecules, see also Fig. 2). The involvement of the enzymes in different biological processes allows understanding the variety of symptoms and features that, with variable frequency, associate with the PRAAS diseases, including immunological, hepatic, splenic, muscular, dermatological, metabolic, hematologic, and cardiovascular abnormalities^12^.

### Polygenic diseases in DAR

DAR includes 264 diseases associated with more than one enzyme (see Fig. 1), 228 of which are also included in Orphanet (https://www.orpha.net/consor/cgi-bin/index.php). Mapping on the Reactome pathways allows to understand whether different enzymes act on the very same biological process or not (see Fig. 3, Polygenic diseases, type A and B, respectively).

**Figure 4:**
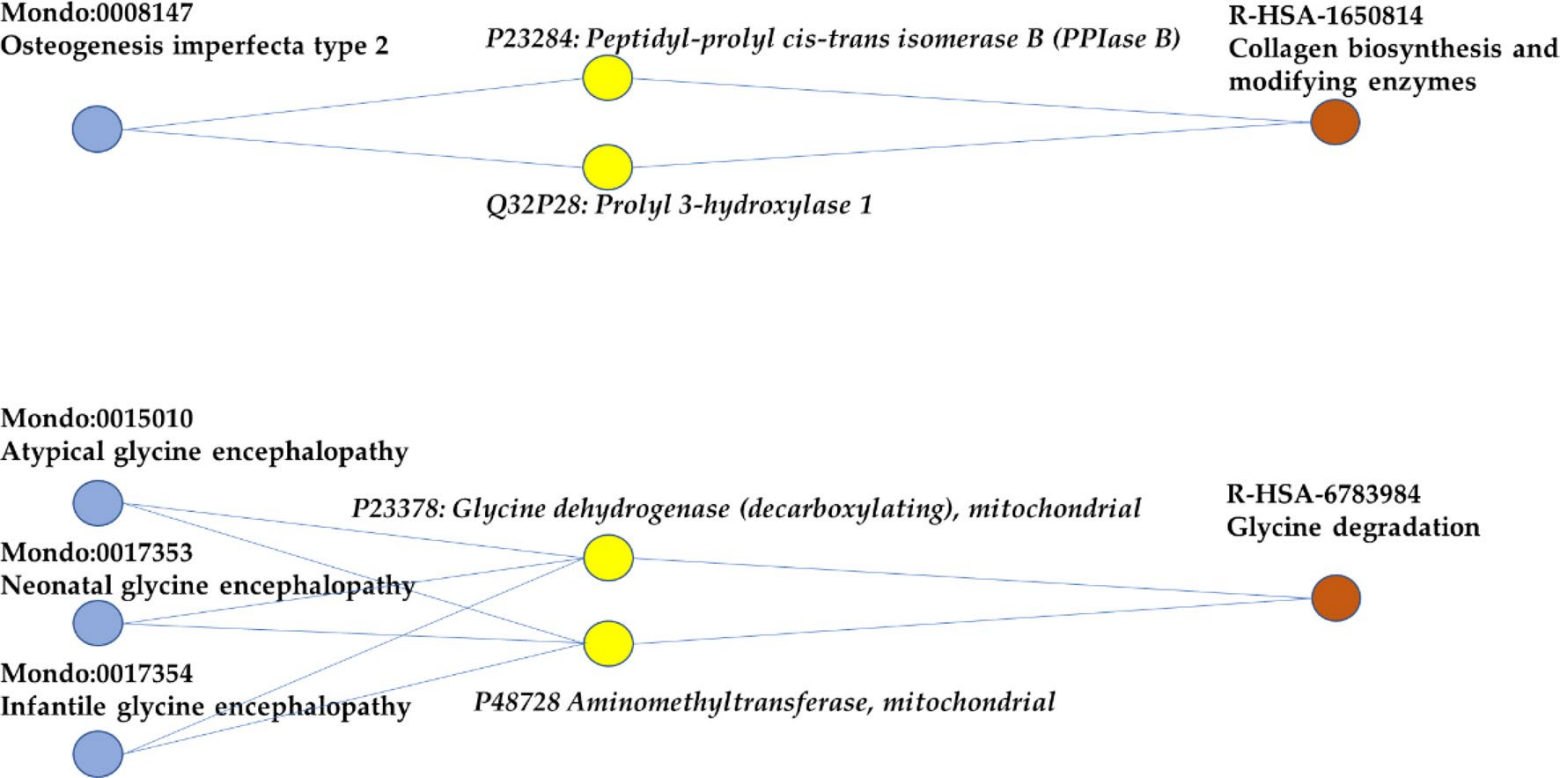
Examples of polygenic diseases associated with only one Reactome pathway (type A in Fig. 3). Blue, yellow and red nodes represent diseases, enzymes, and pathways, respectively.

In 13 cases, the enzymes involved in the same polygenic disease are part of the same pathway (Type A in Fig. 3). Therefore, for this type of diseases, mapping on Reactome explains how different gene defects can lead to the same pathology. Four examples are represented in Fig. 4.

Osteogenesis imperfecta type 2 (Mondo:0008147) is a lethal genetic disorder characterized by increased bone fragility, low bone mass and susceptibility to bone fractures. Variations in two different enzymes have been associated to the disease: the peptidyl-prolyl cis–trans isomerase B (P23284), which catalyses the cis/trans isomerization of peptide bonds involving proline (reaction not detailed in Reactome), and prolyl 3-hydroxylase 1 (Q32P28), catalyzing the post-translational formation of 3-hydroxyproline in -Xaa-Pro-Gly- sequences in collagens, especially types IV and V (Reactome reaction R-HSA-1980233). Both are part of the Reactome R-HAS-1650814 (Collagen biosynthesis and modifying enzymes) and essential reactions for the maturation of collagen, a major component of bones^13^. It is worth noticing that three non-enzyme genes (and therefore not directly present in DAR, although downloadable directly from the Reactome link) are also associated with osteogenesis imperfecta type 2; all are part of the same pathway, two as substrates (Collagen alpha-1(I) chain, P02452 and Collagen alpha-2(I) chain, P08123) and one as auxiliary protein (Cartilage-associated protein, O75718).

The second example in Fig. 4 refers to three similar diseases, Atypical glycine encephalopathy (Mondo:0015010), Neonatal glycine encephalopathy (Mondo:0017353), and Infantile glycine encephalopathy (Mondo:0017354). The three diseases are also known as non-ketotic hyperglycinemias and they are due to alterations of the glycine metabolism that lead to the accumulation of glycine in body fluids and tissues, including the brain, and result in neurometabolic symptoms of variable severity. The three diseases are associated with two mitochondrial enzymes, the decarboxylating glycine dehydrogenase (P23378, EC:1.4.4.2) and the aminomethyltransferase (P48728, EC:2.1.2.10), that are both part of the Reactome pathway R-HAS-6783984 (Glycine degradation)^14^.

Most polygenic diseases are associated with more than one Reactome pathway (Fig. 3, Polygenic- Type B), with complex links between genes and pathways.

**Figure 5:**
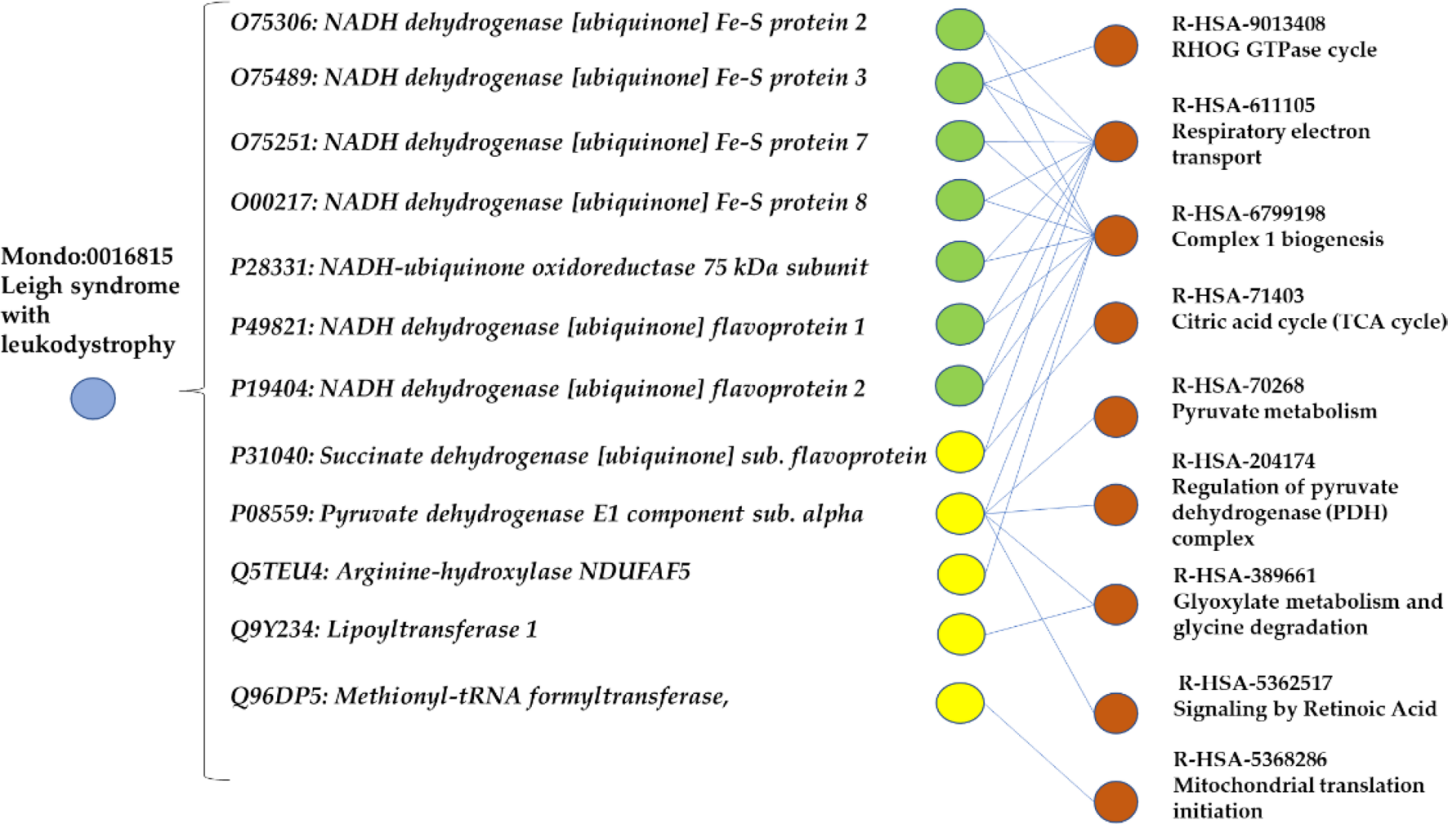
An example of a polygenic disease associated with many Reactome pathways (Polygenic disease-Type B in Fig. 3). The blue dot represents the disease (Leigh syndrome with leukodystrophy) associated to 12 different enzymes (in green enzymes that are part of the mitochondrial complex 1 and in yellow other enzymes). Red nodes represent the Reactome leaf pathways associated with the enzymes.

Figure 5 shows the example of the Leigh syndrome with leukodystrophy (Mondo:0,016,815), a severe disorder resulting from defective mitochondrial energy generation characterized by neurodegeneration (specifically the degeneration of the brain white matter, and possible multisystemic effects on the cardiac, hepatic, gastrointestinal, and renal organs).

The syndrome is associated with 29 mitochondrial genes, 12 of which encode for enzymes (Fig. 5). Collectively they participate into 9 different Reactome pathways. Ten genes belong to the complex 1 biogenesis pathway (R-HSA-67991998), and eight of these are involved in the respiratory electron transport pathway (R-HAS-611105). In particular, seven are subunits of the mitochondrial complex 1 (in green, in Fig. 5)^15^.

### Disease groups in the enzyme-disease-Reactome mapping

**Figure 6:**
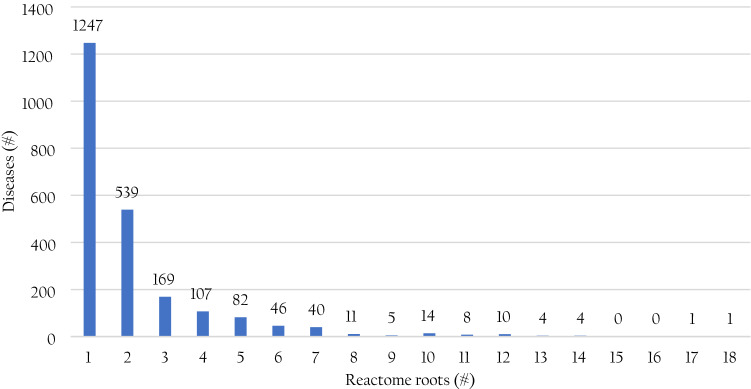
Distribution of diseases with respect to the Reactome roots they are associated with. #: number of. The number of enzymes in each group is reported at the top of each bar. The extreme cases are the colorectal cancer (Mondo: 0005575) and the hereditary breast carcinoma (Mondo:0016419) associated with 18 and 17 roots, respectively.

By expanding the Reactome hierarchy, diseases associate to the 27 Reactome roots. The majority of diseases (1247) univocally map on one root only (Fig. 6), while the remaining 1041 are associated to more than one root, with the extreme cases of colorectal cancer (Mondo:0005575) and hereditary breast carcinoma (Mondo:0016419) associated with 18 and 17 roots, respectively.

The distribution of diseases across the Reactome roots is described in Table 1S. The column “All diseases” refers to all the diseases that map on a specific root, independently of the number of associated roots, while the column “Single root associated diseases” considers only the univocally associated 1247 diseases. The most populated root is “Metabolism”, both when considering all (989 diseases) or univocal (560 diseases) associations; 56.6% of the disease mappings to the “Metabolism” root are univocal. This is the highest percentage across the roots, with the only exception of the scarcely populated “Digestion and absorption”. For all other roots, associated diseases have a more complex mapping on the different processes described by the Reactome hierarchy. The extreme cases are the roots “Autophagy”, “Cell–cell communication”, “Reproduction”, and “DNA replication” that lack univocally associated diseases.

**Figure 7:**
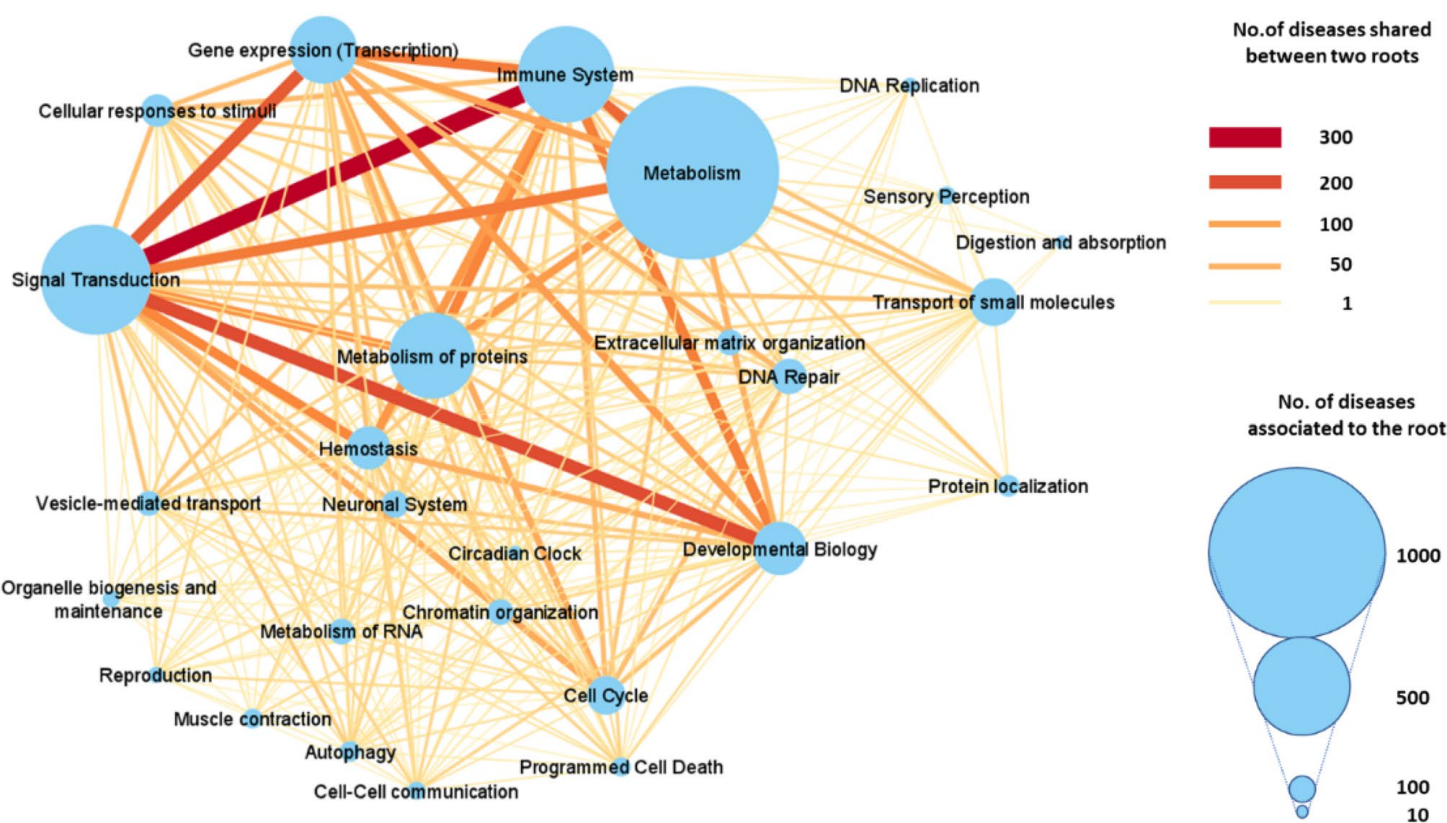
Disease Groups and their interactions. Network representation of the Reactome roots shared by the diseases in DAR. Each node represents a Reactome root, and its radius is proportional to the total number of diseases that associate to the root. Links represent the diseases that share Reactome roots. The wider and redder the link, the higher the number of connecting diseases. Diseases mapping in n (n ≥ 2) Reactome roots are counted for anyone of the possible n(n-1)/2 pairs. Cytoscape (https://cytoscape.org/) was used for the graphical representation.

The patterns of Reactome roots shared by diseases is complex and can be depicted with the network in Fig. 7. The 27 roots are represented with circles whose radius is proportional to the total number of diseases that map on them. Out of the 351 possible links among the 27 nodes (27 × 26/2), 278 are actually present. It appears that the number of diseases shared between two nodes (as represented by the width and the colour of the link) is not simply proportional to the number of the diseases (as represented by the radius of the nodes). The most connected pairs are: “Signal transduction” and “Immune system” (279 diseases), “Signal transduction” and “Developmental biology” (203 diseases), and “Signal transduction” and “Gene expression” (188 diseases), highlighting the centrality of Signal transduction in the network connecting the Reactome roots.

To quote one example of the complexity, the root “Circadian Clock” includes 7 enzymes collectively associated in DAR with 18 diseases (9 monogenic and 9 polygenic). Only one enzyme (Serine/threonine-protein kinase SIK1, EC:2.7.11.1, P57059) is univocally associated with the “Circadian clock” root and it is involved in one monogenic disease (developmental and epileptic encephalopathy 30, Mondo:0014595). The other six enzymes are part of at least another Reactome root besides “Circadian clock”, and the Histone acetyltransferase p300 (EC: 2.3.1.48, Q09472) is the extreme case, participating to 11 Reactome roots. The complexity of association increases for polygenic diseases. This is the case of colorectal cancer (Mondo:0005575), associated with 18 enzymes, one of which is included in the “Circadian clock” root (Histone acetyltransferase p300). Interestingly, the relation between colorectal cancer and processes involved in circadian clock has been recently highlighted^16^.

## Conclusions

Why to include Reactome for understanding human genetic diseases associated to enzymes?

In the present paper we are keen to include in one database most of the information for a gene, and particularly his environment. In DAR, taking advantage of the data included in Reactome from the Gene Expression Atlas (https://www.ebi.ac.uk/gxa/home) it is possible for a given gene in a given pathway to retrieve, besides the associated genetic disease/s, its location, the level of expression of the genes in the pathways in different tissues, and the reaction/s they catalyse.

We implemented a resource which helps focusing for each disease on all the enzymes (and molecules) that the Reactome pathway highlights, including the level of expression of the different molecules participating into the pathway. This can help in better planning experiments for elucidating the possible role of candidate genes in the disease development, considering that our database/toolkit is to be taken as a preliminary investigation for its potentiality in rendering a better glimpse of the disease complexity, even in cases where traditionally the relationship was considered of the one gene-one disease type.

The problem of how to tackle the disease universe has been addressed before and two major concepts emerged in the postgenomic era: (1) the concept of disease modules^17–19^, (2) the concept of comorbidity that is quite often under-appreciated given the paucity of molecular data presently available^17,20^.

A recent paper by adopting multiscale network analysis derived rare disease signatures across multiple levels of biological organization^18^.

Here, we like to add to this scenario starting from disease features that are directly derived from biochemical and molecular biology data for each single gene, and included in organized modules of the cell biological process, such as those of Reactome.

We focus our investigation on human enzymes and the genetic diseases they are linked to.

By mapping genes into Reactome pathways and then associating diseases to genes, following the Monarch initiative, we can produce a disease/s-enzyme gene-Reactome pathways association. This offers a new perspective in the scenario of the disease-gene association, since it allows detailing several associations among diseases, biological processes and molecules involved. Indeed, our procedure highlights that monogenic and polygenic diseases can include either one or more Reactome pathways (Fig. 3) and we offer examples of these different categories. This procedure is novel and helps in dissecting the complexity of all the possible associations among diseases and their molecular mechanisms. Furthermore, diseases can be grouped according to Reactome roots (presently 27, Table 1S and DAR) and by this we can establish links through disease nodes for studying possible co-morbidities (Fig. 7).

All our data are publicly available in DAR, at https://dar.biocomp.unibo.it.

## Supplementary Information

Supplementary Information.

## Data Availability

The dataset generated and analyzed in the current study is available at the DAR database: https://dar.biocomp.unibo.it.
