# Supplementary material for: Mapping human disease-associated enzymes into Reactome allows characterization of disease groups and their interactions
Source: Sci Rep. 2022 Oct 26;12:17963. doi: 10.1038/s41598-022-22818-5 (PMC9605996; doi:10.1038/s41598-022-22818-5)
Supplement: Supplementary file 1 — Supplementary Information. [file 41598_2022_22818_MOESM1_ESM.docx]

**Supplementary Materials**

Mapping human disease-associated enzymes into Reactome allows characterization of disease groups and their interactions.

*DAR: a database of human genetic disease-enzyme-Reactome pathway associations*


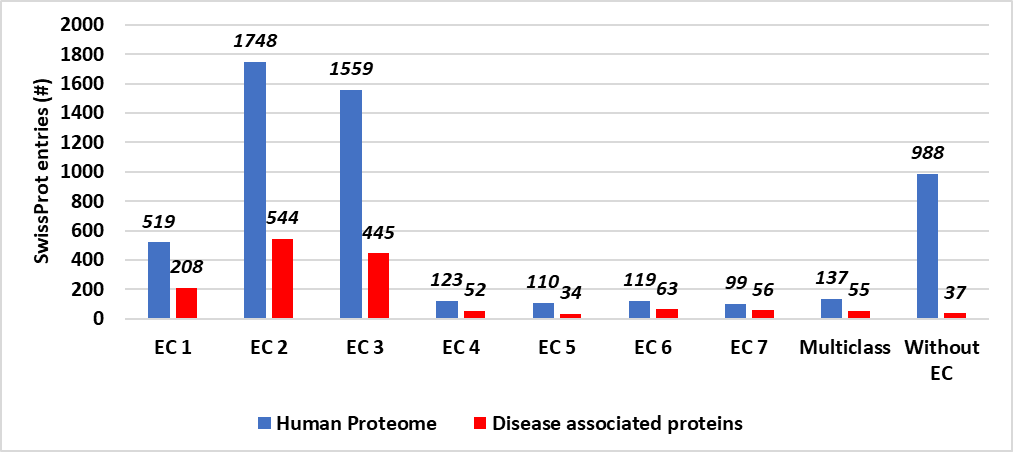


**Figure 1S.** Distribution of enzymes across Enzyme Commission functional classes. #: number of. The number of proteins is reported at the top of each bar. SwissProt includes 5402 human enzymes (in blue) and the dataset of disease associated proteins comprises 1494 enzymes (in red). Multiclass: enzymes endowed with two or more EC level 1 classifications. Without EC: enzymes annotated with the GO term “Catalytic activity” and not endowed with an EC classification.


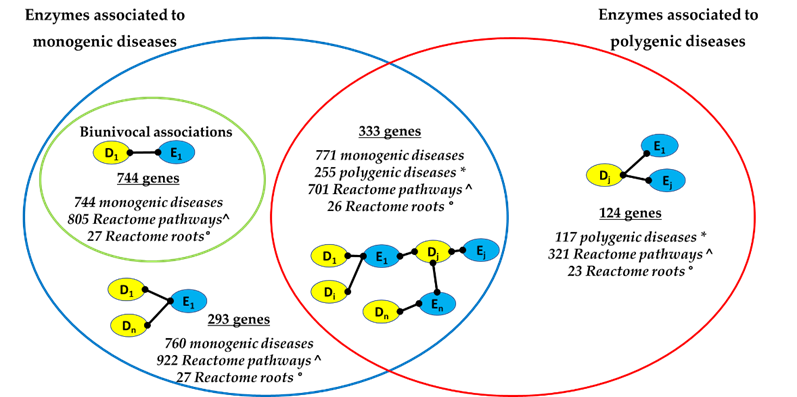


**Figure 2S**. Disease associations of the 1494 enzymes in DAR. Blue and yellow nodes represent enzymes (E) and diseases (D), respectively. * 333 genes are associated to both monogenic and polygenic diseases in 701 Reactome pathways. ^ the same Reactome term can appear in more than one list; ° the same Reactome root can appear in more than one list.

When considering genetic diseases associated to enzymes in our data set, four sets are possible. Within monogenic diseases (enclosed in the blue circle), 744 are biunivocally associated to 744 enzymes, which map into 805 Reactome pathways (and in 27 Reactome roots), and 293 genes are univocally associated to 760 diseases and 922 Reactome pathways. As to polygenic diseases (in the red circle of Fig.3), 124 genes are associated to 117 diseases in 321 Reactome pathways. The intersection contains 333 genes which are associated to 771 monogenic and 255 polygenic diseases, respectively, and mapped into 701 Reactome pathways . This gene-centric view is useful to understand the content of our DAR which allows to search for each gene and all the possible known relationships.


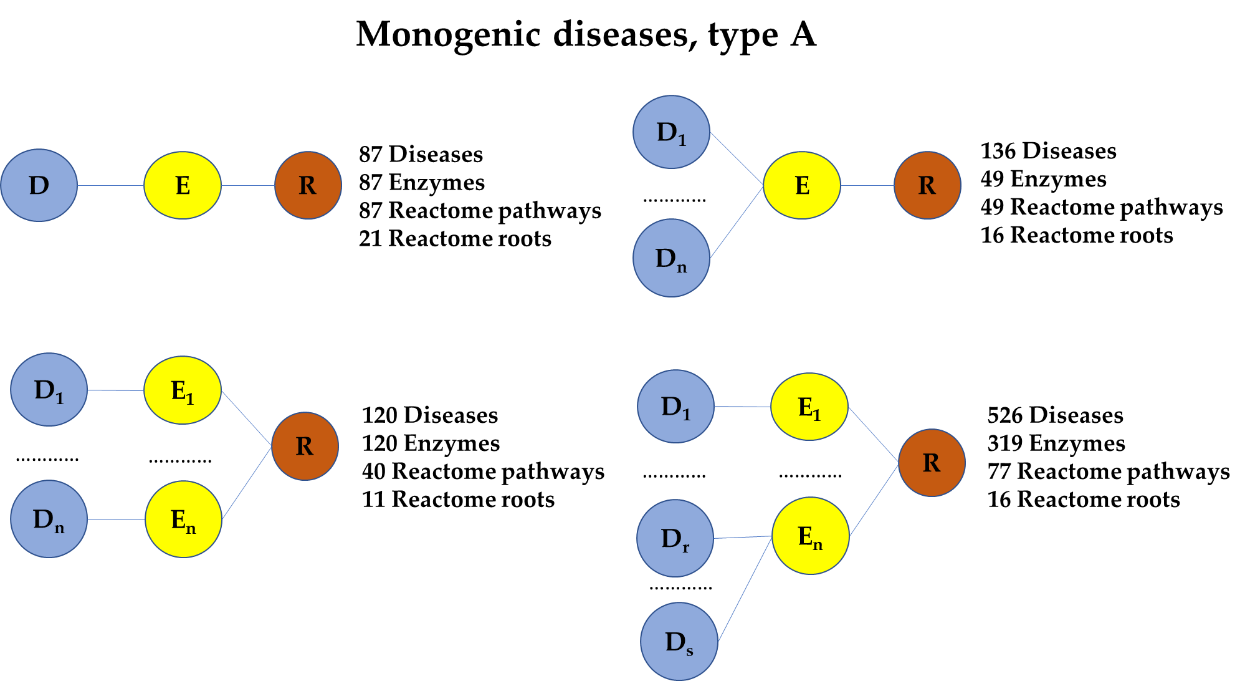


**Figure 3S**. Dissecting the complexity of genetic monogenic diseases based on Reactome pathways. D=Disease; E=Enzyme; R=Reactome leaf pathway

**Table 1S.** Distribution of diseases across Reactome roots

| **Reactome root** | **All diseases**  **#diseases *#enzymes*** | **Single root associated diseases**  **# diseases (%diseases)***  ***#enymes*** |
| --- | --- | --- |
| R-HSA-1430728: Metabolism | 989 *600* | 560 (56.6%) *399* |
| R-HSA-162582: Signal Transduction | 603 *252* | 104 (17.2%) *50* |
| R-HSA-168256: Immune System | 520 *241* | 73 (14.0%) *52* |
| R-HSA-392499: Metabolism of proteins | 455 *231* | 178 (39.1%) *112* |
| R-HSA-74160: Gene expression (Transcription) | 342 *129* | 20 (5.8%) *9* |
| R-HSA-1266738: Developmental Biology | 255 *85* | 23 (9.0%) *10* |
| R-HSA-382551: Transport of small molecules | 220 *103* | 75 (34.1%) *41* |
| R-HSA-109582: Hemostasis | 193 *62* | 5 (2.6%) *3* |
| R-HSA-1640170: Cell Cycle | 166 *73* | 19 (11.4%) *9* |
| R-HSA-73894: DNA Repair | 145 *60* | 37 (25.5%) *22* |
| R-HSA-8953897: Cellular responses to stimuli | 129 *63* | 15 (11.6%) *11* |
| R-HSA-112316: Neuronal System | 94 *38* | 4 (4.2%) *4* |
| R-HSA-8953854: Metabolism of RNA | 86 *52* | 35 (40.7%) *26* |
| R-HSA-4839726: Chromatin organization | 86 *42* | 17 (19.8%) *13* |
| R-HSA-1474244: Extracellular matrix organization | 87 *51* | 35 (40.2%) *25* |
| R-HSA-5653656: Vesicle-mediated transport | 81 *34* | 4 (4.9%) *3* |
| R-HSA-9609507: Protein localization | 62 *34* | 3 (4.8%) *3* |
| R-HSA-9612973: Autophagy | 54 *12* | 0 (0%) *0* |
| R-HSA-397014: Muscle contraction | 49 *19* | 6 (12.2%) *3* |
| R-HSA-5357801: Programmed Cell Death | 46 *18* | 7 (15.2%) *3* |
| R-HSA-9709957: Sensory Perception | 41 *21* | 10 (24.4%) *6* |
| R-HSA-1500931: Cell-cell communication | 40 *6* | 0 (0%) *0* |
| R-HSA-1852241: Organelle biogenesis and maintenance | 32 *13* | 7 (21.9%) *3* |
| R-HSA-1474165: Reproduction | 26 *10* | 0 (0%) *0* |
| R-HSA-69306: DNA Replication | 20 *13* | 0 (0%) *0* |
| R-HSA-400253: Circadian Clock | 18 *7* | 1 (5.5%) *1* |
| R-HSA-8963743: Digestion and absorption | 11 *7* | 9 (81.8%) *5* |

Percentage refers to the number of univocally associated diseases with respect to the total number of diseases associated with the Reactome root
